# Enhancing Oxygen Evolution Reaction Performance with rGO/CoNi-Prussian Blue-Derived Oxyhydroxide Nanocomposite Electrocatalyst: A Strategic Synthetic Approach

**DOI:** 10.1021/acsami.4c09452

**Published:** 2024-09-26

**Authors:** Pedro H. S. Borges, Josué M. Gonçalves, Carmel B. Breslin, Edson Nossol

**Affiliations:** † Institute of Chemistry, Federal University of Uberlândia, 38400-902 Uberlândia, MG, Brazil; ‡ Mackenzie Institute for Research in Graphene and Nanotechnologies (MackGraphe), Mackenzie Presbyterian Institute, 01302-907 São Paulo, SP, Brazil; § Department of Chemistry, Maynooth University, Maynooth W23 F2H6, Co. Kildare, Ireland; ∥ Kathleen Lonsdale Institute, Maynooth University, Maynooth W23 F2H6, Co. Kildare, Ireland

**Keywords:** electrocatalysis, nanocomposite, Prussian blue analogue, graphene, OER

## Abstract

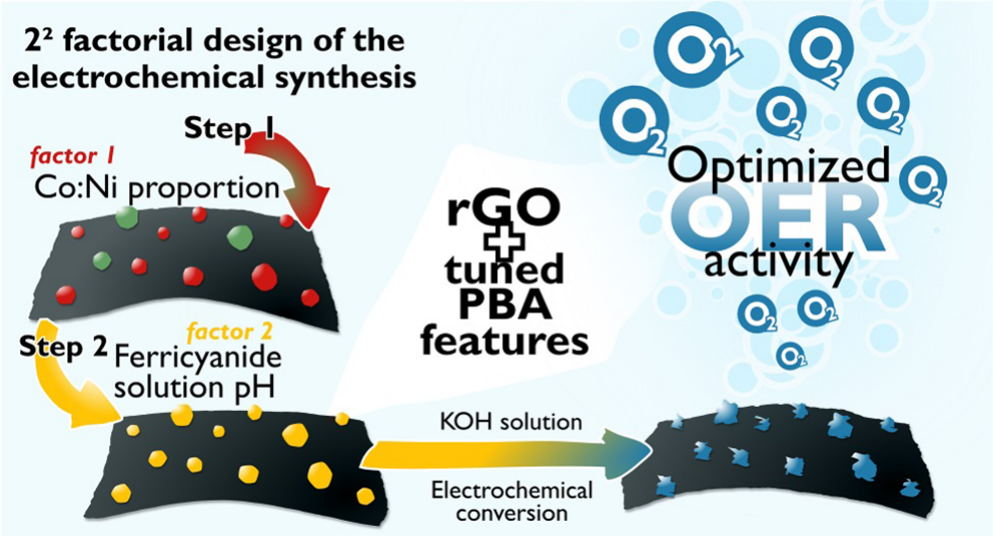

Electrochemical water splitting is a promising approach in the development of renewable energy technologies, providing an alternative to fossil fuels. It has attracted considerable attention in recent years. The benchmark materials used in water splitting are precious metals that are expensive and scarce. Therefore, this work proposes a strategic electrochemical synthesis of a reduced graphene oxide and cobalt–nickel hexacyanoferrate (rGO/CoNiHCF)-derived composite (rGO/CoNiPBd-OOH) to achieve optimized OER performance. The optimum rGO/CoNiHCF was fabricated with the Co:Ni precursors in a 3:1 ratio with a ferricyanide solution of pH = 1.0. Using an alkaline electrochemical treatment, the well-distributed globular particles of CoNiHCF over rGO sheets were converted into layered frameworks of metallic (oxy)hydroxide species, giving the final rGO/CoNiPBd-OOH nanocomposite. This nanocomposite presented favorable kinetic activity resulting in a Tafel slope of 33 mV dec^–1^, while rGO, CoNiPBd-OOH, and RuO_2_ exhibited slopes of 80, 47, and 51 mV dec^–1^, respectively. Although the benchmark RuO_2_ electrocatalyst showed a lower overpotential (240 mV dec^–1^) at a current density of 10 mA cm^–2^, the rGO/CoNiPBd-OOH performed well with an overpotential of 346 mV, combined with superior stability compared to CoNiPBd-OOH and RuO_2_, maintaining a current density of 10 mA cm^–2^ for 15 h with an overpotential loss of 6.92%. This work successfully presents an “all-electrochemical” synthesis of a rGO/CoNiHCF-derived material with remarkable electrocatalytic activity for OER assisted by a strategic preparation methodology, which helped to understand the influence of synthetic parameters and choose their conditions to achieve the optimum OER performance.

## Introduction

The replacement of fossil fuels with eco-friendly energy sources is of great scientific interest, as their cleaner outputs help to protect the environment and address climate targets. Among several options of green energy devices, water splitting exhibits some notable characteristics such as zero pollution emissions, when combined with renewable energy. The overall system of water splitting involves two electrochemical processes: the oxygen evolution reaction (OER) and the hydrogen evolution reaction (HER). While both processes are kinetically sluggish, demanding additional electrochemical potential, the OER has a very high activation energy. One way to reduce the overpotential is to design electrocatalytic materials that are efficient in the electron transfer step and that can facilitate the essential adsorption and deadsorption steps that occur during the OER and HER.^1,2^

The state-of-the-art electrocatalysts for OER and HER are ruthenium and iridium-based oxides, and platinum-based materials, respectively. However, the large-scale application of these precious metals/metal oxides is impracticable due to their high cost and scarcity.^2^ Alternatively, more abundant transition metal-based materials are attracting considerable attention as potential OER and HER electrocatalysts.^3^ Specifically, nickel, cobalt, and iron mixed compounds are interesting and these can be derived from Prussian blue structures which are easily prepared and cost-effective.^4^

Prussian blue analogues (PBAs), also known as metal hexacyanoferrates (MHCF), are polynuclear compounds with mixed-valence metal centers octahedrally coordinated by cyano ligands. The general formula AxM′y[Fe(CN)_6_]z·qH_2_O (where A = alkali metal cation, M′ = transition metal, and x, y, z, and q = stoichiometric coefficients) demonstrates the opportunity to tune the composition and properties according to the final application. These materials can be prepared by coprecipitation, electrochemical, microemulsion, hydrothermal, and solvothermal methods. The choice of the synthesis method, as well as the nature of the transition metal component, is an important strategy to adjust the properties of the material. Accordingly, PBA-based materials are employed in (bio)sensing, biomedicine, adsorption, smart windows, catalysis, and energy storage and conversion.^5,6^

In addition to tunable structures, PBAs possess ordered atomic-level porosity and notable crystallinity, however, they generally present poor conductivity and stability. A common and simple strategy to overcome these issues is to combine the PBA electrocatalysts with carbon-based matrices.^7,8^ For example, Wang and collaborators^9^ studied the influence of the amount of multiwalled carbon nanotubes (MWCNTs) combined with nickel hexacyanoferrate (NiHCF) precatalyst prepared by coprecipitation and assigned the improvement of activity and stability to the presence of the carbonaceous component. Analogously, Lin and colleagues^10^ fabricated functionalized graphene quantum dots (GQDs) containing NiHCF, and using an O_2_-plasma post-treatment observed the same improvement in durability and activity, which was attributed to a synergistic effect between the PBA-derived catalysts and carbon-based materials.

A strategic synthesis route is sustained by a search for adequate compositional and structural features of the OER catalysts, where efficiency indicators, such as activity, stability, and cost, are the main subjects of analysis. A fundamental aspect of the material effectiveness is the number of active catalyst spots.^11^ The increase of this characteristic can be reached by adding high surface area supporting materials, as previously mentioned. Furthermore, the preparation of the derived catalyst using PBA compounds has been shown as a noticeable strategy. The 3D regular zeolitic-like structure of the PBAs generates derived porous structures with easy-reaching active sites, besides their multicompositional aspect, presenting not only metals catalytic sites, but also oxidized carbon and nitrogen moieties that could contribute to the overall OER activity.^12,13^ So, the study of synthetic parameters and their interactions is of high importance for producing efficient and durable catalysts for electrochemical water splitting.

Herein, we propose a simple “all-electrochemical” synthesis of an alkaline OER nanocomposite electrocatalyst based on reduced graphene oxide (rGO) and bimetallic PBA-derived compound (CoNiPBd-OOH). A factorial design of two factors at two levels was employed to understand the impact of the analyzed factors on the material activity and to choose the synthesis parameters that achieve the best OER kinetics. The rGO/CoNiPBd-OOH and its sole components (rGO and CoNiPBd-OOH) were characterized using microscopic and spectroscopic techniques and evaluated as OER electrocatalysts. The rGO/CoNiPBd-OOH exhibited satisfactory OER activity, comparing favorably with the benchmark RuO_2_, and providing an alternative anode material for water splitting cells.

## Experimental Section

### Reagents and Solutions

The solutions were prepared using deionized water. All chemicals were utilized without any purification or treatment. Graphite (200 mesh, 99%), ruthenium(III) chloride (≥99%), and nickel(II) nitrate hexahydrate (≥97%) were acquired from Sigma-Aldrich (USA), while cobalt(II) nitrate hexahydrate (≥98%) and nitric acid (≥65%) were obtained from Synth (Brazil). Trisodium citrate (≥99%), sodium sulfate (≥99%), and potassium nitrate (≥99%) were purchased from Panreac (Spain), Biotec Reagentes Analíticos (Brazil), and Quimex (Brazil), respectively. Potassium ferricyanide (≥99%) was obtained from Êxodo Científica (Brazil) and potassium hydroxide was acquired from Dinâmica (Brazil).

### Instrumentation

Electrochemical measurements were performed in a Squidstat Solo (Admiral Instruments, USA), except for electrochemical impedance spectroscopy (EIS), which was carried out with a PGSTAT204 (Metrohm, Switzerland). X-ray diffraction patterns were acquired in a D8 Advance (Bruker) equipped with a Cu Kα (λ = 0.1542 nm) radiation source and LYNXEYE XE-T detector, operating at 40 kV and 40 mA. Diffractograms were recorded in grazing incidence diffraction (GID) operating mode with a scanning window of 2θ angles of 10–70°, using a scan step of 0.01°. Scanning electron microscopy (SEM) images were obtained by a Vega3 microscope (Tescan, Czech Republic) at 20 kV. Coupled with the SEM microscope, an INCA X-Act X-ray detector (Oxford Instruments, UK) was used for elemental analysis and energy-dispersive X-ray spectroscopy. Fourier-transform infrared (FTIR) spectra were acquired by a Frontier MIR/FIR spectrometer (PerkinElmer, USA) using the attenuated total reflectance (ATR) mode accessory (Pike Technologies, USA). Raman spectroscopy was carried out in a LabRAM HR Evolution microscope (Horiba, Japan) using a 532 nm wavelength with an Ar-ion laser under a potency incidence of 1%.

### Electrochemical Measurements

The electrochemical measurements were acquired in a three-electrode system using a platinum wire, a lab-made Ag_(s)_/AgCl_(s)_/Cl^–^_(sat.)_,^14^ and a glassy carbon (GC) as auxiliary, reference, and working electrodes, respectively. The GC electrode (A_geo_ = 0.071 cm^2^) was vigorously polished in a 0.3 μm alumina aqueous suspension, ultrasonicated in water for 3 min, abundantly washed with water, and dried at room temperature prior to surface modification. For the characterization techniques, the materials were produced over a fluorine-doped tin oxide (FTO) glass electrode. All the calculations and conversions are detailed in the Supporting Information document.

### Preparation of the Electrocatalyst

GO was prepared by a modified Hummers method as described elsewhere.^15^ The synthesis of the nanocomposite electrocatalyst is based on three CV steps and the modified GC electrode was carefully washed with water and dried at room temperature before each step. The first step consists of the simultaneous reduction of GO and metal nitrates over the GC electrode surface, which was carried out by CV in the potential range of −0.5 to −1.6 V at 10 mV s^–1^ for 10 scans. This procedure was performed in a dispersion of GO under magnetic stirring containing 1.0 mg mL^–1^ GO, 0.1 mol L^–1^ Na_2_SO_4_, 2.25 mmol L^–1^ sodium citrate, and a previously defined proportion of Ni(NO_3_)_2_·6H_2_O and Co(NO_3_)_2_·6H_2_O. The second step is the derivatization of the reduced metal species decorated onto the rGO matrix into metal hexacyanoferrate particles. This process was performed by cycling between 0 and 1.0 V at 50 mV s^–1^ until the faradaic current reached its maximum value. An aqueous solution of 0.1 mol L^–1^ KNO_3_ and 1.0 mmol L^–1^ K_3_Fe(CN)_6_ was used for this reaction. The final modification of the electrocatalyst was carried out by immersing the rGO/CoNiHCF-modified GC electrode in a 1.0 mol L^–1^ KOH and then cycling at 100 mV s^–1^ between 0 and 0.6 V until stabilization of the generated redox peaks was observed. Monometallic rGO/CoFe-OOH and rGO/NiFe-OOH PBA-derived control composites were prepared by the same route as the composite of interest, although in the absence of Ni^2+^ or Co^2+^ precursors in the first CV step deposition. The preparation of the RuO_2_ benchmarking electrocatalyst was reproduced by a method described elsewhere.^16^

### Optimization of the Electrocatalyst Synthesis

The optimization was planned based on a 2^2^-factorial design of experiments. This method allows the simultaneous effect analysis of the factors on the studied response. The design was performed by selecting two factors in the synthesis of the electrocatalyst; (1) the mole fraction of the metallic precursors (Co and Ni) and (2) the pH value of the precursor solution of the second step, i.e., the derivatization to hexacyanoferrate species. The first factor (mole fraction) is denoted as ‘x’ in the cobalt concentration (X_Co_), while the second is named pH_HCF_. The two variables were studied at low (−1) and high (+1) levels: with the mole fractions set at 0.25 (−1) and 0.75 (+1) and the pH value of the ferricyanide solution at 1.0 (−1) and 7.0 (+1). A simple strategy to avoid an excessive quantity of experiments for the estimation of the experimental error is to perform the replicates at the center level of the factors. Therefore, this analysis was performed with the central point of the levels of the factors, i.e., 0.5 for the mole fraction and 4.0 for the pH. This design generated 7 experiments that were randomly run. The response analyzed was the Tafel slope extracted from the OER electrocatalytic region of a LSV in a 1.0 mol L^–1^ KOH solution.

## Results and Discussion

### Optimization of the Nanocomposite Preparation

The optimization of the nanocomposite preparation is based on a 2^2^-full factorial design which generated seven experiments and the Tafel slope of the OER catalytic region (in a 1.0 mol L^–1^ KOH solution by LSV) was taken as the response studied (adjusted R^2^ = 0.9748). The levels, factors, experiments, and responses are summarized in Table 1.

**Table 1:** 2^2^-Full Factorial Design of the Optimization of the Electrocatalyst Preparation

	Level		
Factor	(−1)	(0)	(+1)
**X**_**Co**_	0.25	0.5	0.75
**pH**_**HCF**_	1.0	4.0	7.0

Run	X_Co_	pH_HCF_	Tafel slope/mV dec^–1^
**1**	0.25	1.0	36
**2**	0.75	1.0	33
**3**	0.25	7.0	55
**4**	0.75	7.0	55
**5**	0.5	4.0	50
**6**	0.5	4.0	55
**7**	0.5	4.0	54

**Figure 1:**
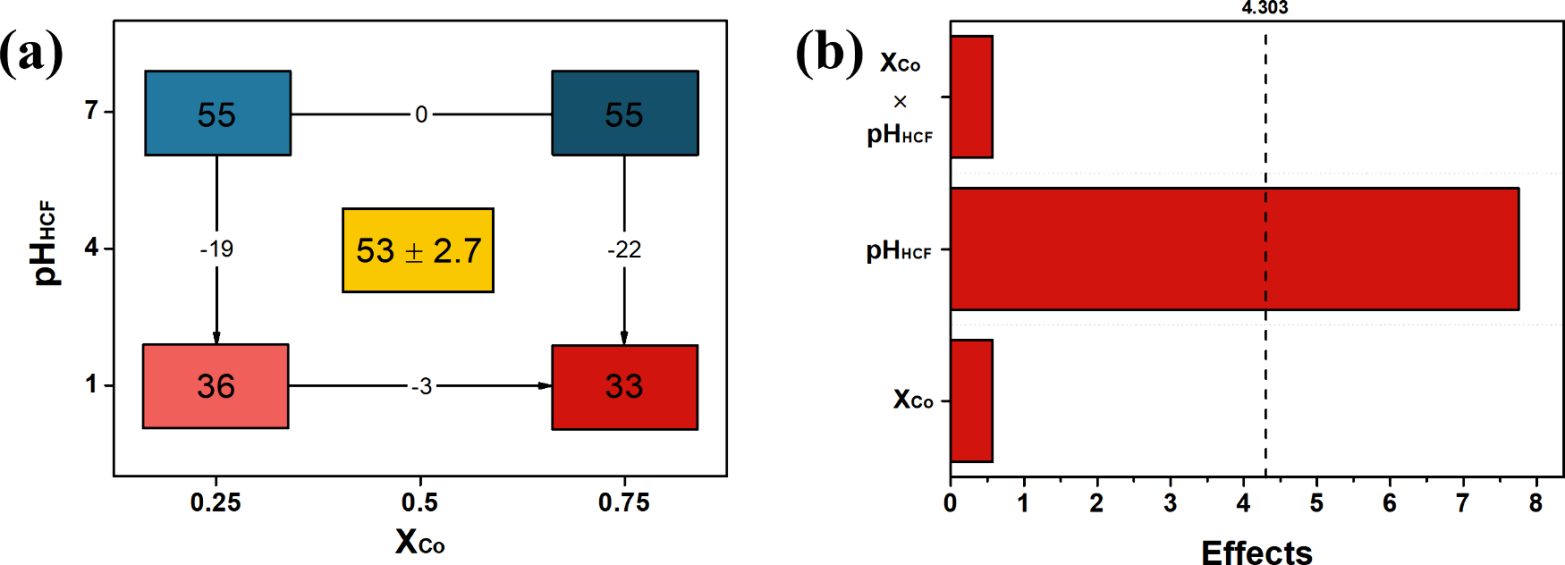
2^2^-full factorial design of the electrocatalyst preparation: (a) geometric representation of the effects (response in mV dec^–1^), and (b) Pareto chart.


Figure 1a exhibits the geometric representation extracted from the experimental design which shows lower Tafel slopes for the lower pH levels employed in the second step of the nanocomposite synthesis. The Pareto chart (Figure 1b) confirms the statistical significance of the pH value variation over the results, while the molar fraction of Co^2+^ and the interaction between the factors do not statistically affect the resulted Tafel slope. A lower Tafel slope indicates more favorable electron and mass transfers.^17^eq 1 demonstrates that a low pH value contributes to a lower response and, although it is not statistically significant, according to the same equation, a favorable contribution of the X_Co_ comes at its higher level. Based on this, experiment 2, Table 1, was chosen to perform the subsequent measurements in this work. The Tafel slopes resulting from the full factorial design of the experiments can be observed in Figure S1.

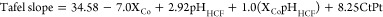
1

### Electrochemical Synthesis

**Figure 2:**
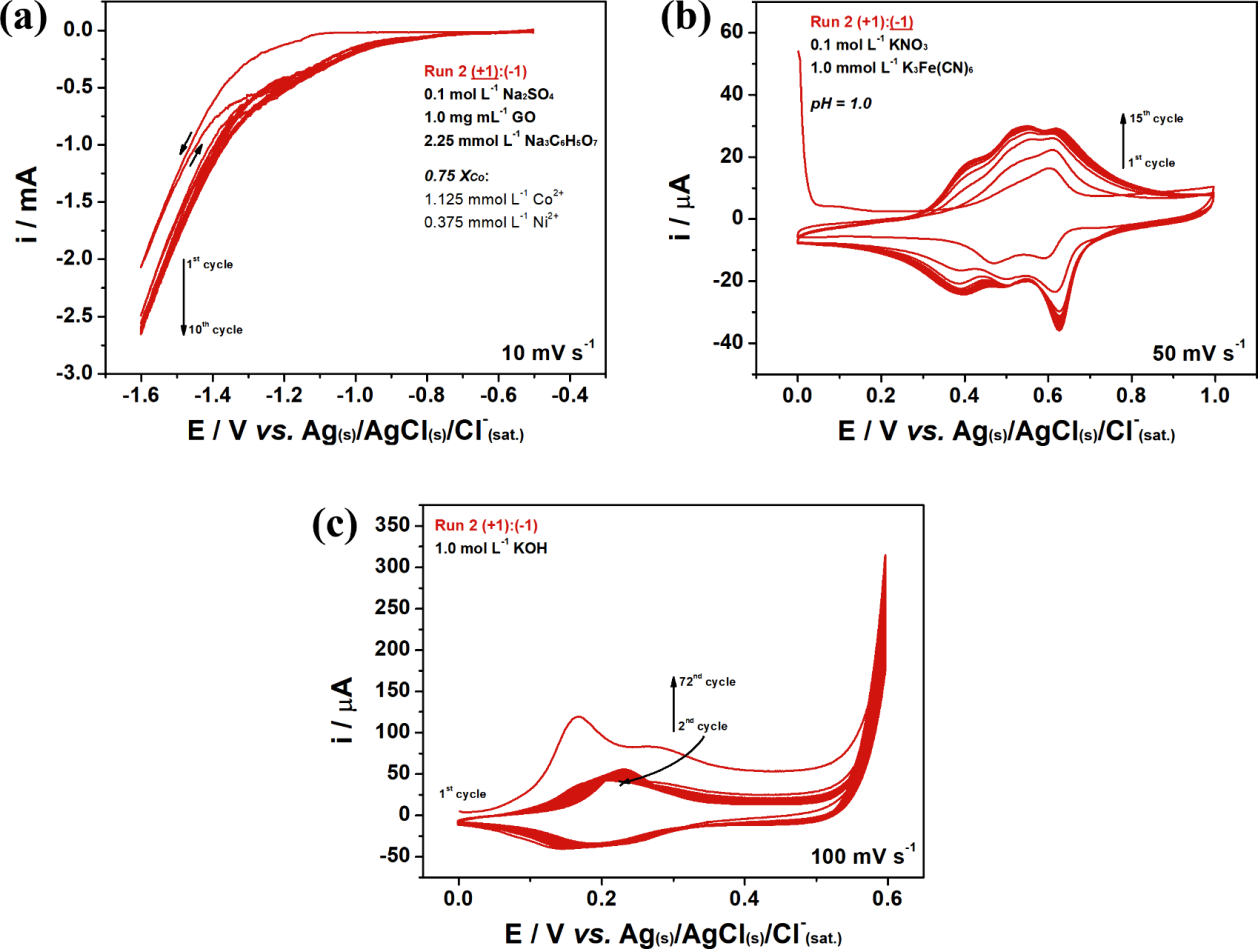
CV results from the synthesis of the chosen nanocomposite electrocatalyst (Run 2): (a) simultaneous reduction of GO and metallic salts (first step); (b) derivatization of the metallic particles into hexacyanoferrate in rGO framework; (c) transformation of the PBA into the electrocatalyst.

The first step of the preparation of the nanocomposite consists of the simultaneous electrochemical reduction of 1.0 mg mL^–1^ GO, 1.125 mmol L^–1^ Co(NO_3_)_2_·6H_2_O, and 0.375 mmol L^–1^ Ni(NO_3_)_2_·6H_2_O, in the presence of 2.25 mmol L^–1^ Na_3_C_6_H_5_O_7_, using 0.1 mol L^–1^ Na_2_SO_4_ as the supporting electrolyte. The procedure was performed by cyclic voltammetry over 10 scans between −0.5 and −1.6 V at 10 mV s^–1^ and the resulting voltammogram can be visualized in Figure 2a.

Three processes can be identified in the CV. At −0.7 V the onset of a clear cathodic current followed by a broad reduction wave is observed, and this is related to the simultaneous electrodeposition of Co and Ni.^18^ At lower potentials of −1.0 V, the irreversible electrochemical reduction of the functional oxygen groups in the graphitic skeleton of GO and its consequential restoration of the sp^2^ framework between carbon atoms is seen. At potentials lower than about −1.3 V, the reduction of water occurs to form hydrogen gas. At this point, there is competition between the HER and the continuous reduction of additional GO functional groups that reduce at more negative potential.^19,20^ Although it seems that the HER hinders GO electroreduction, it is important to apply these potentials to remove the functional groups that are more difficult to reduce. The magnetic stirring that is applied to maintain a consistent dispersion of the GO in the solution phase will also facilitate the removal of the hydrogen bubbles formed at potentials lower than −1.3 V. Furthermore, it is possible to observe that the current intensity enhances with each new scan, which is related to higher amounts of the conducting material at the electrode surface.^21,22^

The second step in the preparation of the material is the conversion of the Co and Ni metallic structures into CoNiHCF through cyclic voltammetry between 0 and 1.0 V at 50 mV s^–1^ in a 0.1 mol L^–1^ KNO_3_/1.0 mmol L^–1^ K_3_Fe(CN)_6_ solution (pH = 1.0) until the current reaches a steady state, which in this case was 15 cycles. The resulting voltammogram is depicted in Figure 2b. The first cycle shows a sudden drop in the current that is related to the anodic redissolution of the immobilized Co and Ni species at the surface of the electrode.^23,24^ It is noticeable that the stripped metallic cations immediately react with the available ferricyanide anions at the electrode/solution interface, and redeposit onto the electrode surface to give the Co and Ni hexacyanoferrate forms as described in Equations S8 and S9.^25,26^ The increase of the current in the subsequent scans indicates the continuous formation of CoNiHCF particles over the rGO surface.^27^

The rGO decorated with monometallic hexacyanoferrates was prepared to compare their CV profiles with the rGO/CoNiHCF and the 15th cycle of their derivatization step are depicted in Figure S2. Clearly, the rGO/NiHCF and rGO/CoHCF show two and three redox pairs, respectively, which are related to electron transitions of the metal sites in the hexacyanoferrate structure. The two redox pairs of the rGO/NiHCF profile, which present higher reversibility due to the low Δ*E*_p_, are related to the Fe^II/III^ transitions in two different forms of the PBA and are represented by eqs 2 and 3.^27^ The rGO/CoHCF CV profile shows three apparent redox pairs. The first two are related to the electron transition of Co^II/III^ (*E*_pa_ = 403 mV and *E*_pc_ = 380 mV) and Fe^II/III^ (*E*_pa_ = 549 mV and *E*_pc_ = 486 mV) and are represented by eqs 4 and 5. The third redox pair (*E*_pa_ = 625 mV and *E*_pc_ = 611 mV) may be associated with a transition of Fe^II/III^ that is located in a distinct stoichiometric environment that is different to the environment giving rise to the second redox pair. This phenomenon is related to the synthesis method that uses metallic species as precursors to derivatize them into hexacyanoferrates and it is described elsewhere.^28,29^ The CV profile of the rGO/CoNiHCF is similar to that of rGO/CoHCF, which was expected, as the concentration of the Co is three times higher than Ni in the rGO/CoNi precursor. However, it is also possible to observe that the position and magnitude of the peaks are somewhat different for the rGO/CoNiHCF and rGO/CoHCF, indicating that the presence of Ni in the structure affects the mass and electron transfer of the composite, proving that the bimetallic hexacyanoferrate has a different structure than the monometallic systems.^30,31^
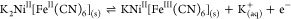
2
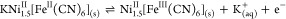
3
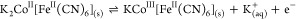
4
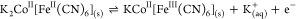
5

The final step of the rGO/CoNiPBd-OOH electrochemical synthesis consists of the derivatization of the rGO/CoNiHCF in 1.0 mol L^–1^ KOH by CV between 0 and +0.6 V at 100 mV s^–1^. The maximum current and steady-state conditions were seen at 72 cycles, and the resulting voltammograms can be visualized in Figure 2c. The first cycle presents an irreversible anodic peak at 168 mV that is associated with the oxidation of the Co species along with the anionic exchange of NO_3_^–^ to OH^–^. The following cycles gradually increase until stabilization, which indicates the saturation and complete structure transformation. The only resulting apparent redox activity presented by the rGO/CoNiPBd-OOH-modified electrode is associated with the conversion of metal hydroxide spots into oxyhydroxide that are responsible for driving the catalytic evolution of oxygen. eq 6 demonstrates the redox characteristics of the metallic structure while Figure S3 exhibits the mechanistic pathway of OER in CoNiPBd-OOH-based compounds.^1,32,33^
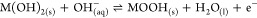
6

### Characterization

#### X-ray Diffraction

The crystalline nature of the rGOCoNiPBd-OOH and its precursors at each synthesis step were analyzed by XRD and the resulting diffractograms are depicted in Figure S4. All materials showed high-intensity peaks associated with the FTO substrate, which pattern is characterized by the tetragonal phase of SnO_2_ (JCPDS #41–1445).^34^ The first step of synthesis–a simultaneous GO/metals electroreduction–resulted in a material with characteristic XRD peaks of face-centered cubic arrangements of metallic CoNi at 41.7 (100) and 44.3° (111) (JCPDS #01–1259 and #04–0850 for Co and Ni, respectively). Also, it is possible to observe a small peak at 40.7° (100) that is associated with hexagonal close pack metallic structures,^35,36^ suggesting a successful electrochemical reduction of CoNi-mixed-phase metallic structures.

The rGOCoNi precursor after electrochemical derivatization in ferricyanide solution resulted in the PBA composite rGOCoNiHCF. It is confirmed by the XRD peak matching with JCPDS #14–0291, which assigns to a face-centered cube phase found in the majority of the hexacyanoferrate-type materials.^37^ The final step and activation of the rGOCoNiPBd-OOH catalyst is performed by CV of rGOCoNiHCF in alkaline media, which suggests the process oxidizes its framework into a different compositional species with distinct arrangement. The thin film exhibited only two observable weak peaks at 31.7 and 39.0° that could be attributed to an amorphous mixed cobalt–nickel hydroxide α and β phases, which match to JCPDS #48–0083 and #26–0480 cards, respectively. These findings are in accordance with previous publications that detail the conversion of PBA frameworks into amorphous oxyhydroxide layered structures by electrochemical alkaline treatment.^38,39^

#### Cyclic Voltammetry

**Figure 3:**
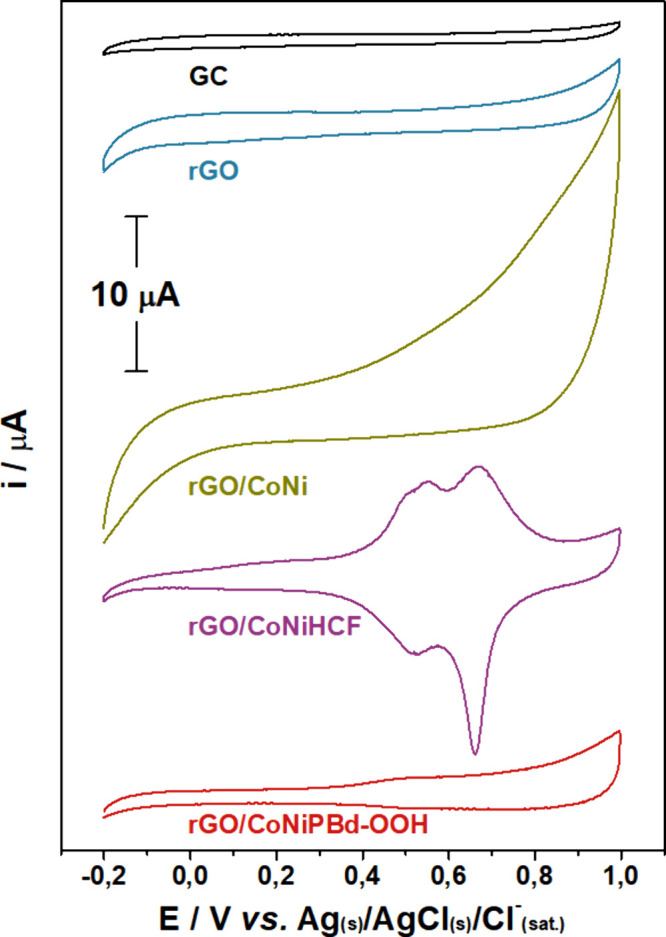
CV profile of different materials prepared in this work at 10 mV s^–1^ in a 0.1 mol L^–1^ KNO_3_ solution.

The materials obtained at each preparation step were characterized by CV at 10 mV s^–1^ in 0.1 mol L^–1^ KNO_3_ and the results are shown in Figure 3. The GC electrode exhibits typical capacitive behavior relative to the migration and adsorption of the supporting electrolyte species on its surface. The rGO-modified GC also shows a capacitive behavior, although it is possible to observe a larger profile that is associated with the increased electroactive surface area of the graphitic material. The rGO/CoNi-modified GC electrode, exhibits an even larger capacitive profile, suggesting the contribution of the metallic nanoparticles and a larger overall area of the material.^42^ Moreover, at potentials higher than +0.4 V, two broad anodic peaks start to evolve and they can be assigned to the oxidation of the metallic species in the rGO framework.^43,44^

The CV generated for the rGO/CoNiHCF exhibits two apparent redox pairs at around 533 and 664 mV in this neutral solution. However, in highly acidic media, three peaks are observed, as shown in Figure S2, indicating that the composition of the supporting electrolyte has a significant influence. In Figure S2, the high concentration of protons competes with the K^+^ diffusion into the structure, while in Figure 3, the rGO/CoNiHCF CV is almost entirely influenced by the intercalation of the K^+^ ions. These processes are complex in mixed hexacyanoferrate compounds.^45,46^ According to previous reports, the peak observed at 533 mV can be associated with the oxidation and reduction of the Co and Ni sites, while the redox behavior of the Fe sites is seen at the higher potential (664 mV). Both transitions occur accompanied by the intercalation of K^+^ in and out of the structure (Equations S10 and S11).^29,47,48^ The CV of the rGO/CoNiPBd-OOH shows a broad redox pair with *E*_pa_ = 542 mV and *E*_pc_ = 518 mV. This was attributed to the electrochemical conversion of the metal hydroxide species into oxyhydroxide,^12,49^ as illustrated in eq 6.^49−52^

#### Scanning Electron Microscopy and Energy-Dispersive X-ray Spectroscopy

**Figure 4:**
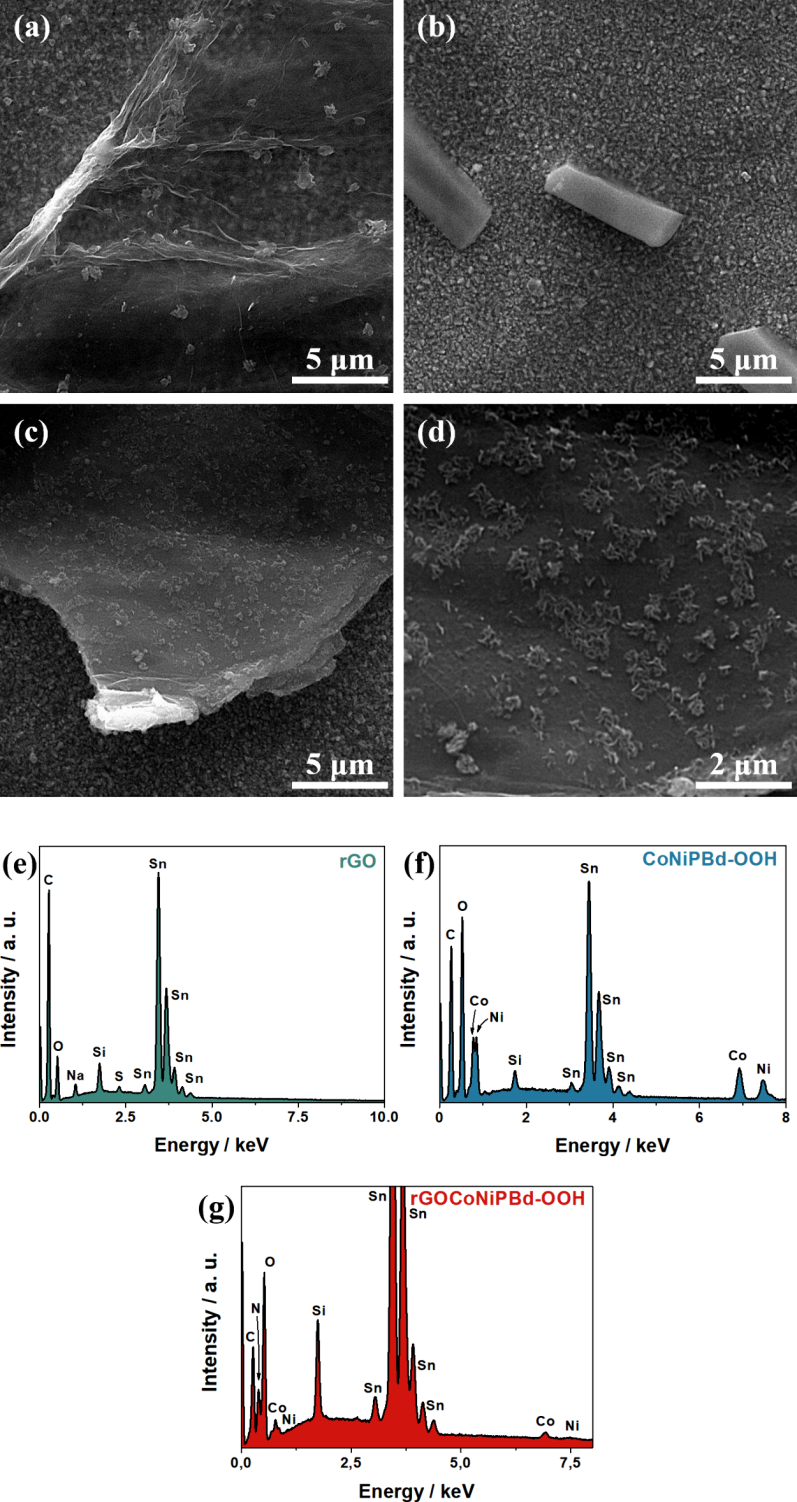
SEM images of the (a) rGO, (b) CoNiPBd-OOH, and (c) and (d) rGO/CoNiPBd-OOH-modified FTO electrodes. EDS elemental analysis spectra of the (e) rGO, (f) CoNiPBd-OOH, and (g) rGO/CoNiPBd-OOH-modified FTO electrodes.

SEM images of the rGO/CoNiPBd-OOH nanocomposite-modified FTO electrode and its constituents separately prepared (rGO and CoNiPBd-OOH) can be visualized in Figure 4. All images exhibited full-covered glass substrates with homogeneous crystalline particles of FTO. Figure 4a shows almost transparent sheets with highly wrinkled edges, typically found in functionalized bidimensional graphitic morphologies. The CoNiPBd-OOH prepared in the absence of rGO demonstrates the tendency to form micrometric rods (Figure 4b), indicating the metal redissolution in acidic ferricyanide solution followed by derivatization in alkaline media promoted particle growth in specific nucleation spots. In contrast, the image of rGO/CoNiPBd-OOH (Figure 4c) shows particles in the nanometric scale with near-layered structures homogeneously anchored over and between the graphitic sheets. It is noticeable that the presence of the carbonaceous material avoids particle agglomeration due to its high surface area and residual functional groups that serve as nucleation sites.^22^ The morphological transformation of the third step can be visualized in Figure S5, which shows drastic changes in the globular-shaped CoNiHCF particles over the rGO, assuming that the alkaline activation promotes particle amorphization in rGOCoNi-OOH, providing more defective sites that contribute for easier interfacial heterogeneous mass transfer on the electrocatalytic OER process.^53^

EDS spectra indicated the elemental composition of the rGO, CoNiPBd-OOH, and rGO/CoNiPBd-OOH-modified FTO electrodes, which can be observed in Figure 4e, f, and g, respectively. All materials exhibited peaks relative to tin (Sn), oxygen (O), and silicon (Si), which constitutes the substrate made of glass with a thin film of FTO. The spectrum of the sole carbonaceous material shows peaks associated with carbon (C) and oxygen (O) that are present in its composition. Moreover, the signals of sodium (Na) and sulfur (S) that show up are derived from residual Na_2_SO_4_ from the supporting electrolyte. The CoNiPBd-OOH produced in the absence of rGO exhibits a spectrum containing cobalt (Co) and nickel (Ni) but does not show the peak related to iron (Fe), which could indicate that this element is present in low amounts or was stripped out of the composite during the derivatization processes. The rGO/CoNiPBd-OOH-modified FTO electrode exhibited all the peaks observed in the other materials in addition to peaks associated with Fe and N, suggesting that the presence of the rGO as supporting material did not only tune the morphology of the catalyst, as seen in SEM images, but can also influence the composition.

#### Fourier-Transform Infrared Spectroscopy

**Figure 5:**
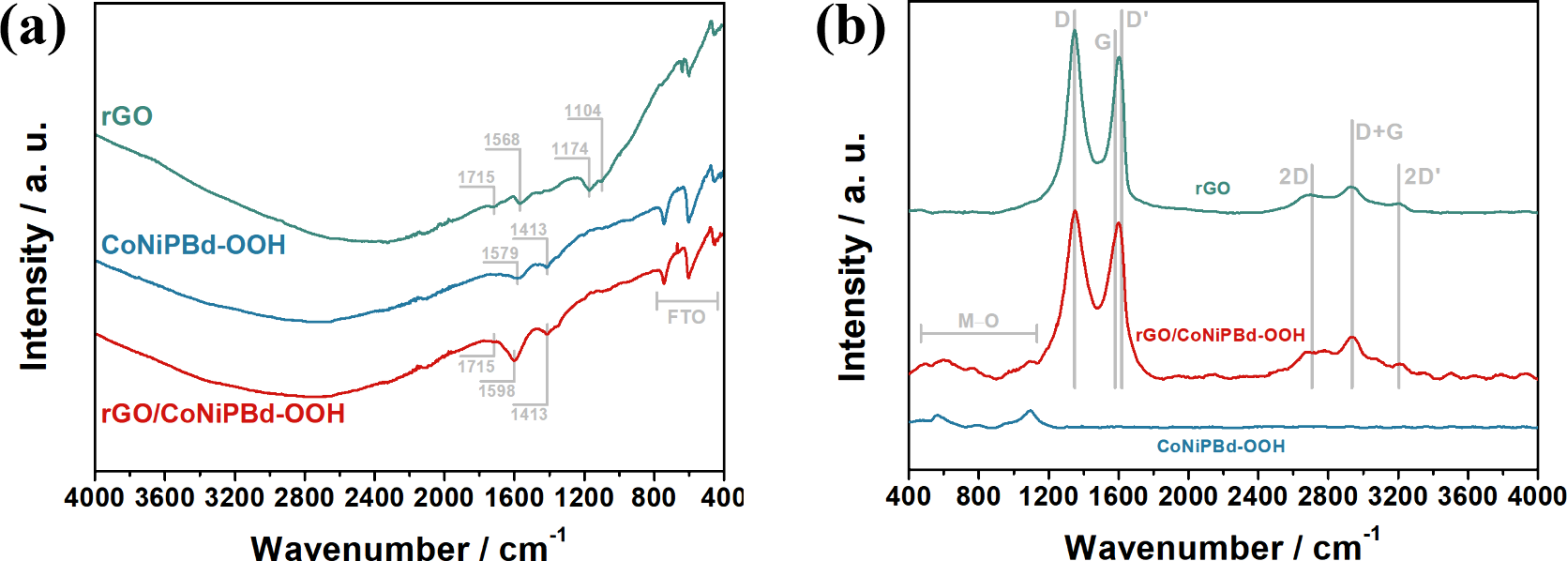
(a) FTIR and (b) Raman spectra of the rGO, CoNiPBd-OOH, and rGO/CoNiPBd-OOH-modified FTO electrodes.

The rGO/CoNiPBd-OOH-modified FTO electrode was characterized by FTIR spectroscopy along with its isolated components and the resulting spectra can be visualized in Figure 5a. All the spectra exhibit three bands between 400 and 800 cm^–1^ assigned to Sn–O vibrations of the FTO structure.^54^Figure S6a shows the FTIR spectrum of GO and rGO to evaluate the effectiveness of the electrochemical reduction in the graphitic structure. The prepared GO from the Hummers modified method, exhibits six clear bands. The broad bands between 3000 and 3600 cm^–1^ are related to stretching modes of hydroxyl groups from water molecules adsorbed in the framework. Also, bands associated with stretching modes of oxygen functional groups in the graphitic framework, such as epoxy, hydroxyl, and carbonyl can be observed at 973, 1049, and 1717 cm^–1^, respectively. Moreover, bands assigned to bending modes of hydroxyl groups related to alcohol/phenol (1356 cm^–1^) and to adsorbed water molecules (1610 cm^–1^) were detected. It is possible to observe a drastic intensity reduction of the majority of the bands after the GO is submitted to the electrochemical reduction. The rGO spectrum shows residual carbonyl groups related to the band at 1715 cm^–1^. Also, the band at 1104 cm^–1^ is associated with the stretching vibrations of C–O generated by the reduction of carbonyl groups. At 1174 cm^–1^ there is a clear band that is assigned to C–O–C stretching vibrations of the remaining epoxy groups that are too stable to be reduced by the electrochemical method. Furthermore, the removal of several hydroxyl groups permitted the observation of the band related to the stretching mode of C=C bonds (1568 cm^–1^) present in the core structure of graphitic materials.^19,55,56^

The spectrum of CoNiPBd-OOH shows two apparent bands at 1413 and 1579 cm^–1^ (Figure 5a). As indicated by the presence of carbon and oxygen in its EDS spectrum (Figure 5b), it is possible to infer that the FTIR signals are associated with symmetric and asymmetric stretching of bonds present in the carboxylate anions (COO^–^). These may originate from the citrate which was used during the electrochemical reduction of the metallic species. Alternatively, the presence of carbon may be connected with the alkaline hydrolysis of the PBA cyano groups into carboxyl.^57−59^ The band corresponding with the C=O vibration at 1715 cm^–1^, associated with the rGO, is also seen in the rGO/CoNiPBd-OOH spectrum.^1^ Also, signals similar to those seen in the CoNiPBd-OOH spectrum are present, indicating the coexistence between the two materials in this composite. It is possible to observe a more prominent band at 1598 cm^–1^, which could be related to the overlapping contribution of carboxylate and graphitic bands. Figure S6b shows the spectrum of the rGO/CoNiHCF precursor and it shows bands related to the stretching vibrations of C ≡ N at 2087 cm^–1^, Fe–CN–M at 598 cm^–1^, carboxylate at 1414 and 1609 cm^–1^ along with carbonyl signal at 1725 cm^–1^ and overlapped graphitic stretching vibration band around 1577 cm^–1^, arising from the rGO framework.^60,61^ These results confirm the complete electrochemical conversion of the bimetallic hexacyanoferrate.

#### Raman Spectroscopy

Raman spectra of the materials can be visualized in Figure 5b. The G band (around 1580 cm^–1^) originates from the sp^2^-hybridized carbon atoms arranged as rings and/or chains in the skeletal framework and it is present in every graphitic structure. The D band, located at 1350 cm^–1^, is only generated by lattice asymmetries induced by the presence of defects. Another important band, namely 2D, is a second order overtone vibration that is sensitive to the number and stacking of monolayers.^62,63^ Additional information is summarized in Table S1 along with the bands positions. The analysis of the ratio between the integrated areas of the D and G bands allows the numeric evaluation of the degree of defects (A_D_/A_G_) and provides the possibility to estimate the lateral crystallite size (L_a_) through Equation S12. Furthermore, the A_D_/A_G_ ratio is also useful to infer the density of defects (η_D_) and the average distance between them can be calculated using Equations S13 and S14.^64^ The A_2D_/A_G_ ratio can provide information about the number of stacked graphene monolayers in the material and their quality regarding crystallinity. A_2D_/A_G_ ratios higher than 2 indicate a monolayer graphene, while values around 1 suggests the presence of a bilayer graphene. Results between 0.5 and 1.0 are consistent with graphene with three layers and around 0.5 is multilayer graphene. This value tends to zero when it is related to an infinite number of layers, typical of graphite structure.^63^ Moreover, through Equation S15, it is possible to obtain the average length of continuous graphene, which provides the estimation of the tortuosity (L_eq_) of the graphitic structure, considering the curves and crumples originated by the defective oxygen-functionalized graphene derivatives.^65^ The shoulder band that appears close to the G band is called D′ and, like the D band, is only active in the presence of defects, and the ratio between the defective-related bands, A_D_/A_D′_, is useful to estimate the types of defects present in the graphitic structure.^66^ The A_D_/A_D′_ ratio that reaches a maximum of 13 is assigned to sp^3^-type defects in the graphitic framework. When the ratio is close to 7, the sp^2^-carbon atoms structure possesses vacancy-type defects, while the minimum A_D_/A_D′_ = 3.5 is related to graphene edges and boundaries.

All materials produced in this work that have a graphitic component achieve an A_D_/A_D′_ ratios close to 7, which indicates graphitic structures with vacancy-like defects. The rGO-based materials that were submitted to the electrochemical reduction showed a slightly lower A_D_/A_D′_ ratio than the nonreduced GO, but were similar for all materials, suggesting the reduction method had the same effect on functional groups in the absence and the presence of the metallic species. Figure S7a exhibits the Raman spectra of rGO and its precursor, GO. The former possesses a higher A_D_/A_G_ ratio of 3.74 compared to the latter (2.78), indicating that the electrochemical reduction process removed oxygen functional groups and at the same time it restored smaller-sized sp^2^ carbon domains.

The A_D_/A_G_ ratios obtained for rGO/CoNiPBd-OOH (Figure 5b) and rGO/CoNiHCF (Figure S7b) are closer to the values obtained for GO, suggesting that the presence of the metallic ions in the simultaneous electrochemical reduction interferes in the elimination of the functional groups but does not affect the restoration of the sp^2^ carbon framework. As the lateral crystallite size and defect density are directly proportional to the A_D_/A_G_ ratio, their values follow the same trend, Table S1. The A_2D_/A_G_ ratio of 0.52 for the GO precursor is consistent with its multilayered structure. All rGO-containing materials showed a slightly higher value, indicating that the electrochemical reduction gives fewer monolayers. Additionally, all the materials showed a L_eq_ > L_a_, consistent with the wrinkled and curvy monolayered structures. In addition to the rGO-based bands, the Raman spectrum of rGO/CoNiHCF shows two additional bands located at 2110 and 2161 cm^–1^. These signals are associated with the vibrations of the cyano ligands in the Fe^II^–C ≡ N–M^II^ and Fe^III^–C ≡ N–M^II^ environments, respectively.^67^

#### Evaluation of rGO/CoNiPBd-OOH Electrocatalyst toward OER

**Figure 6:**
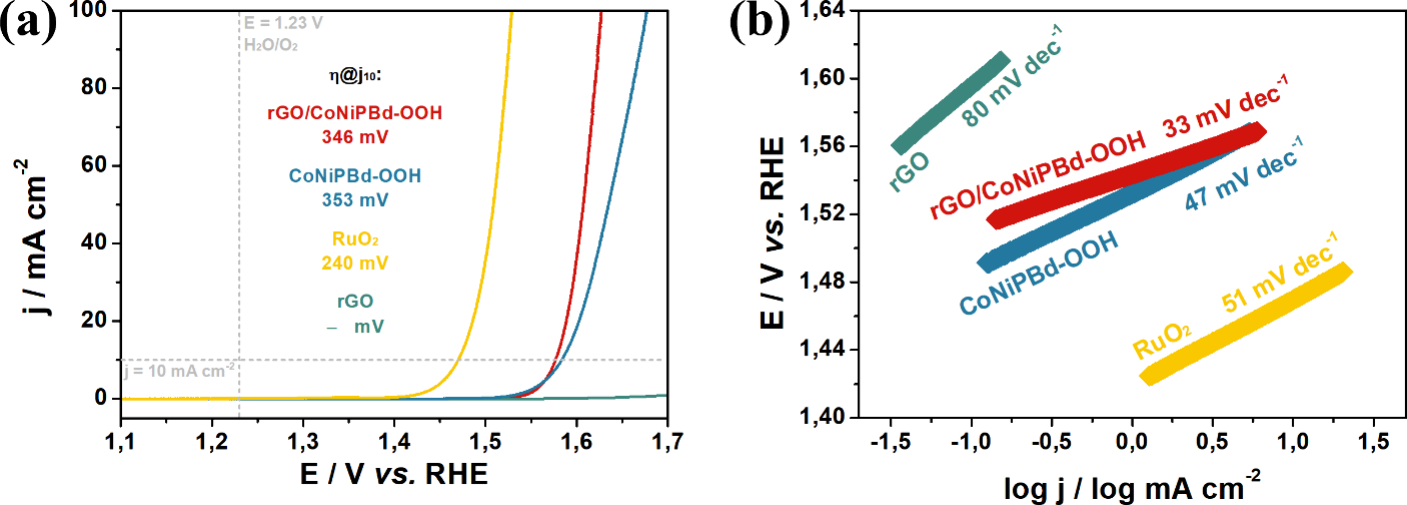
(a) LSV in 1.0 mol L^–1^ KOH solution at 5 mV s^–1^ and (b) Tafel slope of the OER electrocatalysis region promoted by the materials.

The OER performance of the rGO/CoNiPBd-OOH-modified GC electrode was evaluated using LSV at 5 mV s^–1^ in a 1.0 mol L^–1^ KOH solution along with rGO, CoNiPBd-OOH, and RuO_2_-modified GC electrodes for comparison (Figure 6a). The overpotential required to reach *j* = 10 mA cm^–2^ (*j*_10_) was employed in the analysis of the data. The RuO_2_ exhibited the lowest overpotential of 240 mV at *j*_10_, followed by the rGO/CoNiPBd-OOH at 346 mV and CoNiPBd-OOH at 353 mV. Although the rGO has very poor OER activity, its contribution as a supporting matrix for the CoNiPBd-OOH nanoparticles is positive as it reduces the overpotential at *j*_10_ from 353 to 346 mV. The Tafel slopes are summarized in Figure 6b. Even though RuO_2_ presented a lower overpotential, it is noticeable that the rGO/CoNiPBd-OOH possesses superior electrocatalysis kinetics, resulting in a lower Tafel slope of 33 mV dec^–1^ compared to the ruthenium-based catalyst (51 mV dec^–1^). In addition, the presence of the carbonaceous material provides the nanocomposite with enhanced OER kinetics, as the CoNiPBd-OOH, in the absence of rGO, results in a Tafel slope of 47 mV dec^–1^.

In order to compare the synergistic effect of the Co–Ni coexistence in the precatalyst composition, monometallic PBA-derived composites were prepared and had their OER performances evaluated. Figure S8a illustrates the LSV in 1.0 mol L^–1^ KOH solution of the bimetallic rGO/CoNiPBd-OOH compared with monometallic rGO/CoPBd-OOH and rGO/NiPBd-OOH modified GC electrodes and it revealed a lower overpotential to achieve the same 10 mA cm^–2^ of current density. The electrocatalytic activity is also enhanced, as seen in the Tafel slope results of Figure S8b. The bimetallic PBA-derived compound exhibited facilitated OER kinetics in comparison to the monometallic ones, justified by lower Tafel slope values. Singhal and collaborators affirmed that the presence of Ni in the Co lattice induces the formation of a higher amount of surface oxygen sites, which promotes easier interfacial heterogeneous contact of the hydroxyl anions with the active electrocatalyst surface.^68^

**Figure 7:**
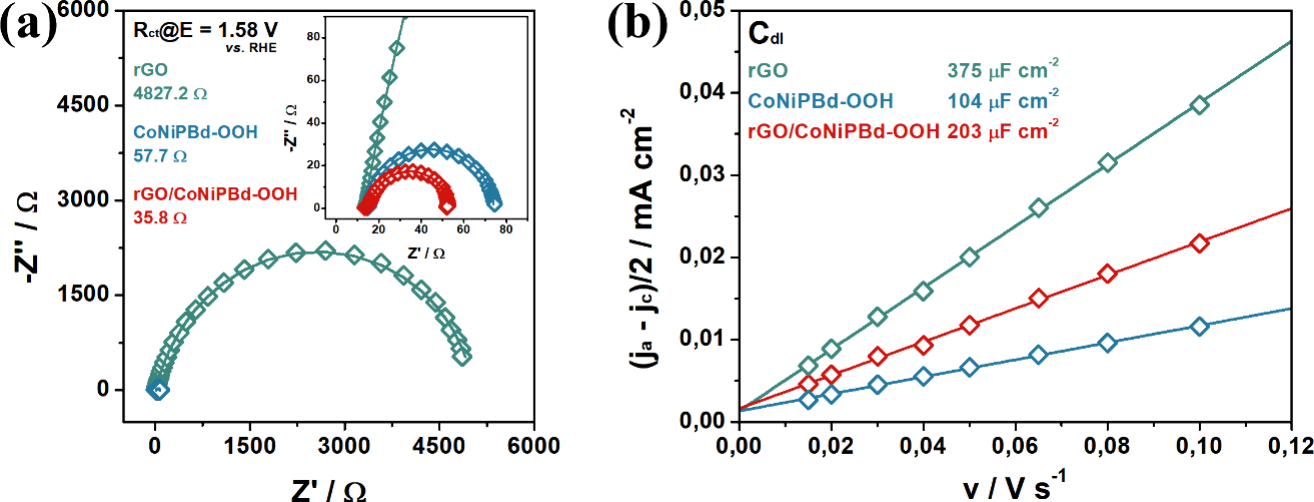
(a) EIS Nyquist diagram of the materials at E = 1.58 V vs RHE and (b) determination of C_dl_ in 1.0 mol L^–1^ KOH solution.

The charge transfer resistance, R_ct_, and impedance of rGO/CoNiPBd-OOH, CoNiPBd-OOH, and rGO were measured using EIS. In Figure Figure 7a Nyquist plots are represented where the data were recorded at 1.58 V vs RHE, while the inset shows the data at the higher frequencies. The equivalent circuit (Figure S9) used in fitting the experimental data consists of two RC couples in series, and the solution resistance, R_S_. The uncompensated resistance of the solution gives an average R_S_ = 13.03 ± 0.09 Ω for all three materials.

The RC couple in the higher frequency region was attributed to the resistance of the electrocatalyst thin film (R_film_).^8,69^ The rGO/CoNiPBd-OOH nanocomposite exhibited the lowest R_film_ of 5.52 Ω, followed by the CoNiPBd-OOH (R_film_ = 36.36 Ω), and rGO (R_film_ = 184.9 Ω) modified electrodes, suggesting the nm sized morphology of the particles allows faster electron transfer in the compact layer of the material. The charge transfer resistance (R_ct_) was computed as R_ct_ = 35.0 Ω for the rGO/CoNiPBd-OOH nanocomposite, while higher values of R_ct_ = 4827.2 Ω and 57.79 Ω, were obtained for the control materials rGO and CoNiPBd-OOH, respectively. This low R_ct_ is consistent with the superior OER of the rGO/CoNiPBd-OOH electrocatalyst.

The electrochemically active surface areas (ECSA) of the materials were estimated using the CV technique. The corresponding CV measurements can be visualized in Figure S10, while the average of the cathodic and anodic capacitive currents (j_a_ – j_c_)/2 is plotted against the scan rate and is shown in Figure 7b. Using linear regression analysis, the double layer capacitances (C_dl_) of the materials, were computed. By dividing C_dl_ by C_s_ (40 μF cm^–2^), the ESCA was estimated. Interestingly, the rGO showed the highest ESCA of 9.4 cm^2^, while the CoNiPBd-OOH had a relatively low ESCA of 2.6 cm^2^, and the rGO/CoNiPBd-OOH nanocomposite returned an intermediary value of 5.07 cm^2^. Although the rGO has the highest ESCA, it is a very poor OER electrocatalyst, however, it does provide the rGO/CoNiPBd-OOH with a higher ESCA compared with the CoNiPBd-OOH. This enlarged surface area and superior OER activity could be related to the nanoparticle morphology and availability of active sites for adsorption and deadsorption of the intermediates, and to a synergistic effect between the carbonaceous material and the PBA-derived species.^8^

**Figure 8:**
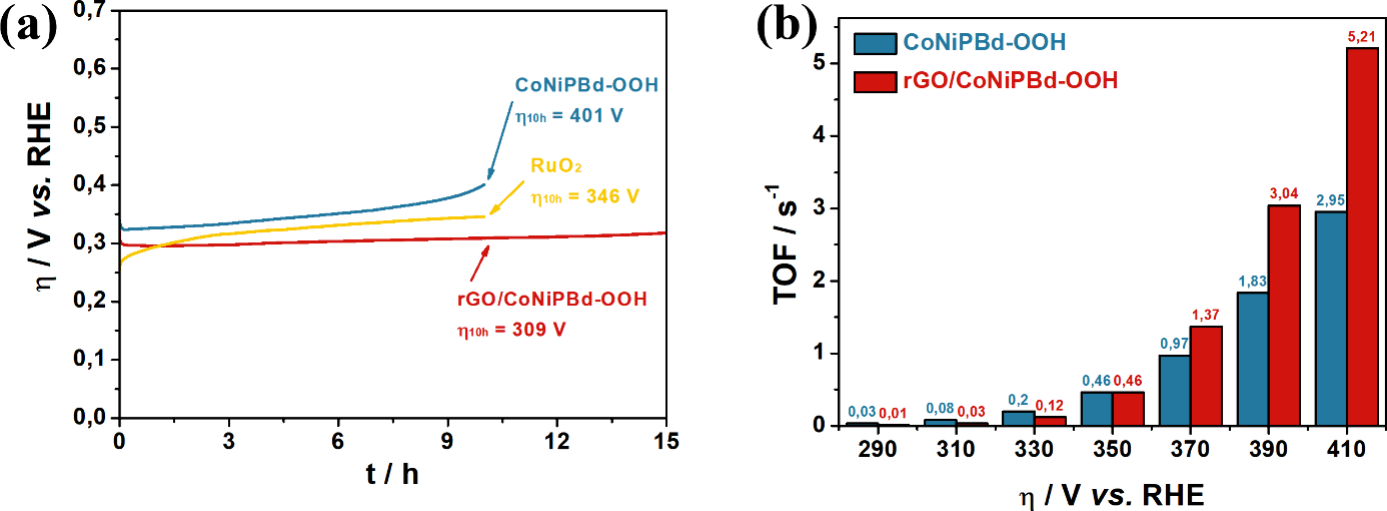
(a) Long-term stability test for rGO/CoNiPBd-OOH and control electrocatalysts and (b) rGO/CoNiPBd-OOH and CoNiPBd-OOH TOF estimation in different overpotential values.

A study on the stability of the rGO/CoNiPBd-OOH electrocatalyst was carried out in a 1.0 mol L^–1^ KOH solution by a potentiometry test at *j*_10_ and its long-term stability was compared to the CoNiPBd-OOH and RuO_2_ electrocatalysts, as illustrated in Figure 8a. The rGO/CoNiPBd-OOH-modified GC electrode showed very good stability over 15 h, resulting in an efficiency loss of 6.92%. After 10 h, the nanocomposite exhibited a minimal loss of 4.23%, which was superior to the CoNiPBd-OOH (19.2%) and benchmark RuO_2_ (26.59%), demonstrating that the carbonaceous component plays a major role in the structural conservation of the electrocatalyst sites in the rGO/CoNiPBd-OOH nanocomposite.^4^Figure S11a shows a slight loss of activity as represented by the higher R_ct_ after 15 h of stability testing, which is also demonstrated by the LSV in Figure S11b, that exhibits a drop of 29 mV at j_10_ in comparison to the LSV of the electrocatalyst prior to the stability test.

The turnover frequency (TOF), which is the estimation of the intrinsic activity of the material at a determined overpotential, is described by the activity per number of active electrocatalytic sites. The number of active sites for rGO/CoNiPBd-OOH and CoNiPBd-OOH-modified GC electrodes were calculated based on the integrated area of the cathodic peak of the electrocatalyst reduction (Figure S12) and the operations are denoted in Equation S6b. The TOF were estimated from 290 to 410 mV vs RHE (Figure 8b) of overpotential. It can be observed that at overpotentials lower than 330 mV, the CoNiPBd-OOH shows high intrinsic activity, suggesting accessibility to the electrocatalyst sites. However, the poorer kinetics at higher overpotentials can be explained by the larger particles with less contact surface. In contrast, the rGO/CoNiPBd-OOH electrocatalyst demonstrates a low intrinsic activity at lower overpotentials. This may be related to the somewhat slow diffusivity of the OH^–^ ions within the multilayered rGO and difficulty in interacting with the active sites. However, once the active sites are accessed, the catalytic activity increases, and at the higher overpotentials, the rGO/CoNiPBd-OOH electrocatalyst exhibits high TOF values of 5.21 s^–1^ at an overpotential of 410 mV.

In Table 2, The OER activity and stability of the rGO/CoNiPBd-OOH composite are compared with several cobalt, nickel, and/or iron-based materials. It is clear from this analysis that the rGO/CoNiPBd-OOH composite has one of the lowest Tafel slopes, good stability and the overpotentials at *j*_10_ compare very favorably with these previously reported electrocatalysts.

**Table 2:** Alkaline Media OER Electrocatalytic Parameters of Several Composites Based on Nickel, Cobalt, and/or Iron over Carbon Structures Presented in the Literature^b^

Material	η@j_10_/mV	Tafel slope/mV dec^–1^	[KOH]/mol L^–1^	Durability@j_10_/h	ref.
**Co**_**0.75**_**Ni**_**0.25**_**Fe**_**2**_**O**_**4**_**/rGO**	440	85	0.1	10	(51)
**Ni@graphene**	370	66	1.0	12	(70)
**Ni–Fe@rGO**	350	38	0.1	5.5	(71)
**Fe**_**3**_**O**_**4**_**@NiS**_**x**_**/rGO-0.04**	330	36	1.0	6	(72)
**CoFe**_**2**_**O**_**4**_**NPs-on-CFP**	378	73	1.0^a^	40	(73)
**NiCoO**_**2**_**/graphene**	436	94	0.1	0.28	(74)
**CoFe/NC**_**30%**_	340	77	1.0	24	(75)
**NiFe25/PGS**	332	33	1.0	10	(76)
**CoFe**_**2**_**O**_**4**_**/gCN/NGQDs**	445	69	1.0	2	(77)
**rGO/CoNiPBd-OOH**	346	33	1.0	15	This work

a NaOH solution.

b **Co**_**0.75**_**Ni**_**0.25**_**Fe**_**2**_**O**_**4**_**/rGO:** Nickel-substituted cobalt–iron oxide supported over reduced graphene oxide; **Ni@graphene:** nickel nanoparticles at graphene; **Ni–Fe@rGO:** Nickel–iron alloy over reduced graphene oxide; **Fe**_**3**_**O**_**4**_**@NiS**_**x**_**/rGO-0.04:** Iron oxide at nickel sulfide over reduced graphene oxide; **CoFe**_**2**_**O**_**4**_**NPs-on-CFP:** Cobalt ferrite nanoparticles on carbon fiber paper; **NiCoO**_**2**_**/graphene:** Nickel cobaltite over graphene; **CoFe/NC**_**30%**_**:** Nickel-substituted cobalt oxide over N-doped carbon; **NiFe25/PGS:** Nickel–iron double layered hydroxide on pyrolytic graphite sheet; **CoFe**_**2**_**O**_**4**_**/gCN/NGQDs:** Cobalt ferrite/graphitic carbon nitride/N-doped graphene quantum dots.

## Conclusions

In this work, a 2^2^-factorial design of experiments, with the OER Tafel slope as the response, was employed to assist in the electrochemical synthesis of rGO/CoNiHCF-derived electrocatalysts for the OER. A strong influence of the ferricyanide solution pH was seen, with a low pH inducing desirable defects in the rGO/CoNiHCF precatalyst structure. The supporting carbon material, rGO, was also essential, providing a high surface area support for the electrocatalytic particles, while facilitating the uniform distribution of the catalyst particles. With this strategic preparation of the rGO/CoNiPBd-OOH nanocomposite, improved stability and activity toward the OER was obtained. This study highlights the possibility to tune and tailor the properties of PBA by varying the relevant synthetic conditions, opening several possibilities regarding the optimization of fabrication methods for the synthesis of nanomaterials.

## Supporting Information Available

General calculations and conversions. Equations employed for electrocatalytic parameters acquisition and PBA formation; Tafel slopes of the experiments performed in the 2^2^-factorial design and CV of rGO/CoHCF, rGO/NiHCF, and rGO/CoNiHCF composites; FTIR and Raman spectra of the precursors and equations applied for carbon-composed materials; OER mechanism, proposed EIS equivalent circuit, and CV of nonfaradaic regions for the materials; CV area integration of the CoNiPBd-OOH-based electrocatalysts and EIS and LSV before and after the 15 h stability test for the rGO/CoNiPBd-OOH composite (PDF)
