# Supplementary material for: Enhancing Oxygen Evolution Reaction Performance with rGO/CoNi-Prussian Blue-Derived Oxyhydroxide Nanocomposite Electrocatalyst: A Strategic Synthetic Approach
Source: ACS Appl Mater Interfaces. 2024 Sep 26;16(40):53705–17. doi: 10.1021/acsami.4c09452 (PMC11472268; doi:10.1021/acsami.4c09452)
Supplement: Supplementary file 1 — am4c09452_si_001.pdf [file am4c09452_si_001.pdf]

Enhancing Oxygen Evolution Reaction Performance with rGO/CoNi-Prussian Blue-Derived Oxyhydroxide Nanocomposite Electrocatalyst: A Strategic Synthetic Approach

Pedro H. S. Borges,[a] *Josué M. Gonçalves,*[b] *Carmel B. Breslin,*[c,d] *Edson Nossol*[a, *]

[a] Institute of Chemistry, Federal University of Uberlândia, 38408-902, Uberlândia, MG, Brazil

[b] Mackenzie Institute for Research in Graphene and Nanotechnologies (MackGraphe), Mackenzie Presbyterian Institute, São Paulo, SP, Brazil

[c] Department of Chemistry, Maynooth University, Maynooth, Co. Kildare, Ireland

[d] Kathleen Lonsdale Institute, Maynooth University, Maynooth, Co. Kildare, Ireland

Supporting information

Electrochemical calculations details

The current density (*j*) was estimated using the geometric surface area, Equation S1. A 1.0 mol L^{-1} KOH solution was used as the electrolyte. The resistance of this solution was estimated using EIS, as $R_s = 13.03 \pm 0.09\ \Omega$ (n = 5). The ohmic drop was 95% compensated using Equation S2. All measurements were carried out against $Ag_{(s)}/AgCl_{(s)}/Cl^{-}_{(sat.)}$ and the reference electrode conversion to the RHE scale was calculated based on Equation S3.

The overpotential (η) was calculated using the thermodynamic potential for the oxidation of water and the formula can be observed at Equation S4. The polarization profiles of the materials were performed by linear sweep voltammetry (LSV) in a 1.0 mol L^{-1} KOH solution at 5 mV s^{-1} between 1.1 and 1.7 V *vs.* RHE and the Tafel slope (b) was extracted from the electrocatalytic region of the LSV using Equation S5. EIS spectra were acquired in a frequency range of 0.1 MHz – 0.1 Hz at a fixed potential of 1.58 V *vs.* RHE under the potential amplitude of 10 mV in a 1.0 mol L^{-1} KOH solution. The electrochemically active surface area (ECSA) was estimated by the double layer capacitance (C_{dl}) through cyclic voltammetry (CV) at increasing scan rates in a region with no faradaic activity (0.82 to 0.92 V *vs.* RHE) in a 1.0 mol L^{-1} KOH solution. The C_{dl} was computed by averaging the anodic and cathodic current densities, $(j_a - j_c)/2$, (note j_c is negative) at 0.87 V *vs.* RHE and plotting this average current against the scan rate. The ECSA, in cm^2, was calculated by dividing the C_{dl} by C_s, that is the specific capacitance of a smooth surface, which is usually taken as 40 μF cm^{-2}. The long-term stability was tested in a 1.0 mol L^{-1} KOH solution at an applied current density of 10 mA cm^{-2}.

The intrinsic activities of the electrocatalysts were evaluated by their turn-over frequency (TOF) at different overpotential values. The TOF was calculated by Equation S6a, where N_a is the Avogadro constant (6.02 × 10^{23} mol^{-1}), *i* is the collected current at that overpotential, *4* is the number of electrons involved in OER, *F* is the Faraday constant (96485 C mol^{-1}), and Γ is the number of electroactive sites. This latter parameter was calculated based on the integrated area of the CV reduction peak of the electrocatalyst in a 1.0 mol L^{-1} KOH solution. The division of the resulting integrated area by the scan rate (v) returns the amount of charge (Q) accumulated. Considering that one electron equates to a charge is 1.602×10^{-19} C, it is possible to estimate Γ, as summarized by Equation S6b.

$$j = \frac{i}{A_{geo}}$$ **Equation S1**

$$E_{compensated} / V = E - (0.95 R_s \times i)$$ **Equation S2**

$$E_{RHE} / V = E_{Ag_{(s)}/AgCl_{(s)}/Cl^{-}_{(sat.)}} + (0.059 \times pH) + 0.197$$ **Equation S3**

$$\eta \,/\, V = E_{RHE,compensated} - 1.23$$ **Equation S4**

$$\eta \,/\, mV = b \times \log j + a$$ **Equation S5**

$$TOF \,/\, s^{-1} = \frac{N_a i}{4F\Gamma}$$ **Equation S6a**

$$\Gamma = \frac{\int i \, dV}{(1.602 \times 10^{-19})\, v}$$ **Equation S6b**

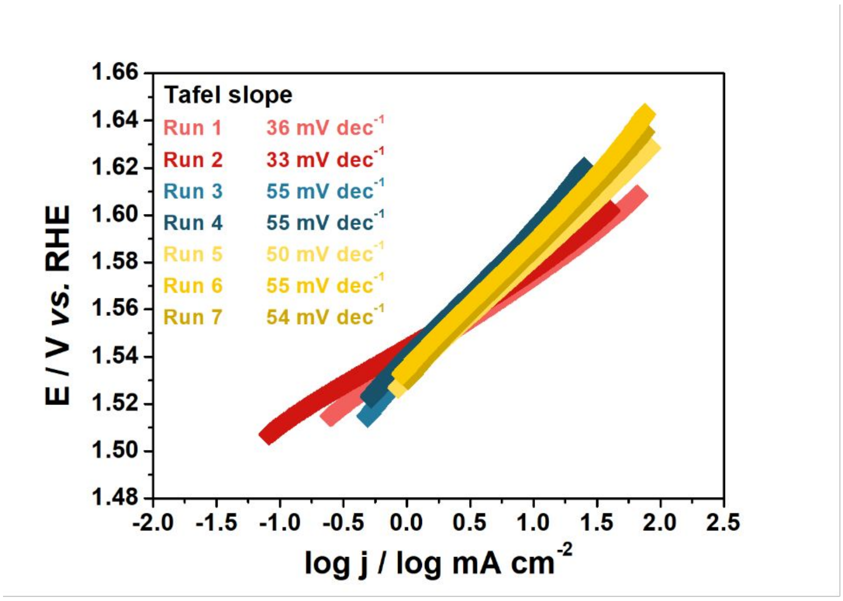

Figure S1. Resulting Tafel slopes extracted from the different experiments performed in the electrocatalysts optimisation.

$$2\ K^{+}_{(aq)} + Co^{2+}_{(aq)} + [Fe(CN)_6]^{4-}_{(aq)} \rightarrow K_2Co[Fe(CN)_6]_{(s)}$$ **Equation S8**

$$2\ K^{+}_{(aq)} + Ni^{2+}_{(aq)} + [Fe(CN)_6]^{4-}_{(aq)} \rightarrow K_2Ni[Fe(CN)_6]_{(s)}$$ **Equation S9**

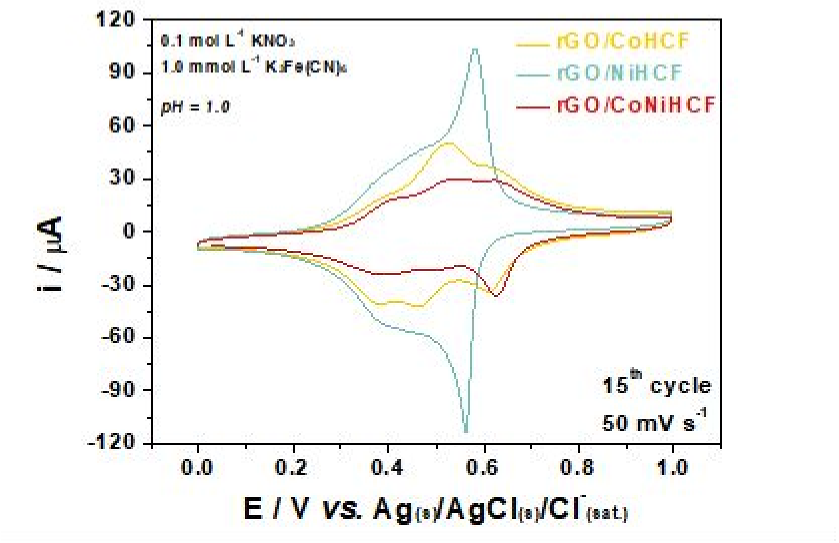

Figure S2. CV 15th cycle of the derivatization of the bimetallic and monometallic hexacyanoferrates/rGO.

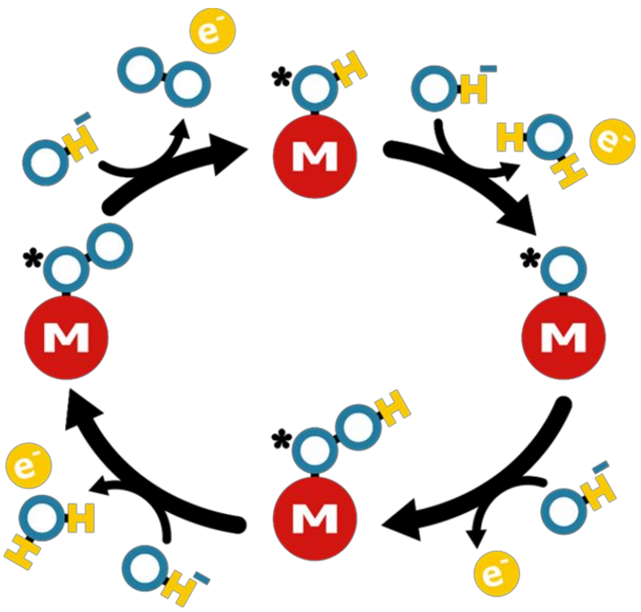

Figure S3. Mechanism pathway for OER in metallic sites.

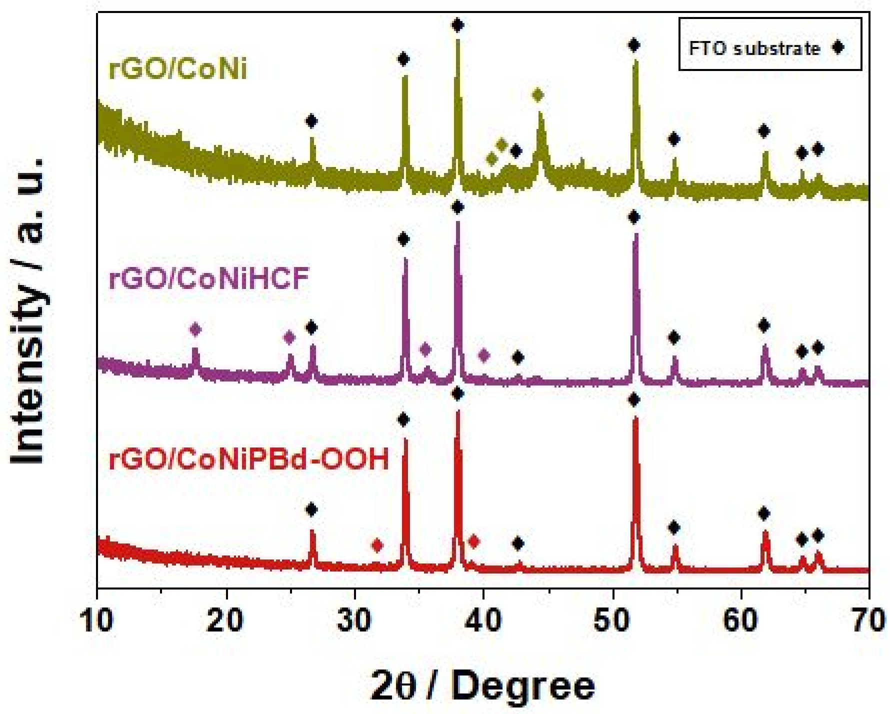

Figure S4. XRD patterns of the materials prepared in the three electrochemical synthesis steps.

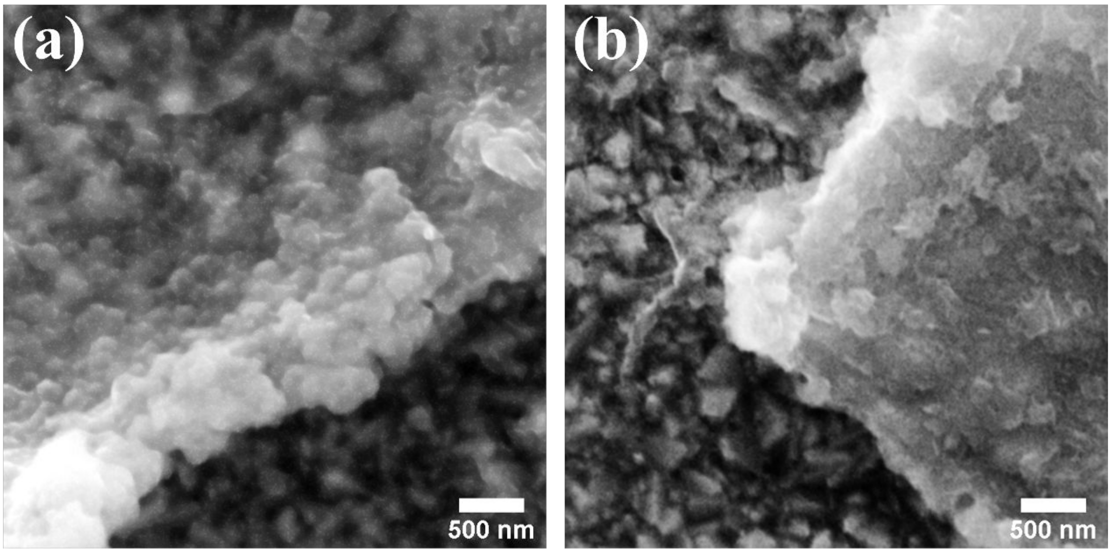

Figure S5. SEM images of **(a)** rGO/CoNiHCF precursor and **(b)** rGO/CoNiPBd-OOH prepared over the same FTO substrate.

$K_2M^{II}[Fe^{II}(CN)_6]_{(s)} \rightleftharpoons KM^{III}[Fe^{II}(CN)_6]_{(s)} + K^+_{(aq)} + e^-$ (M = Co or Ni) **Equation S10**

$KM^{III}[Fe^{II}(CN)_6]_{(s)} \rightleftharpoons M^{III}[Fe^{III}(CN)_6]_{(s)} + K^+_{(aq)} + e^-$ (M = Co or Ni) **Equation S11**

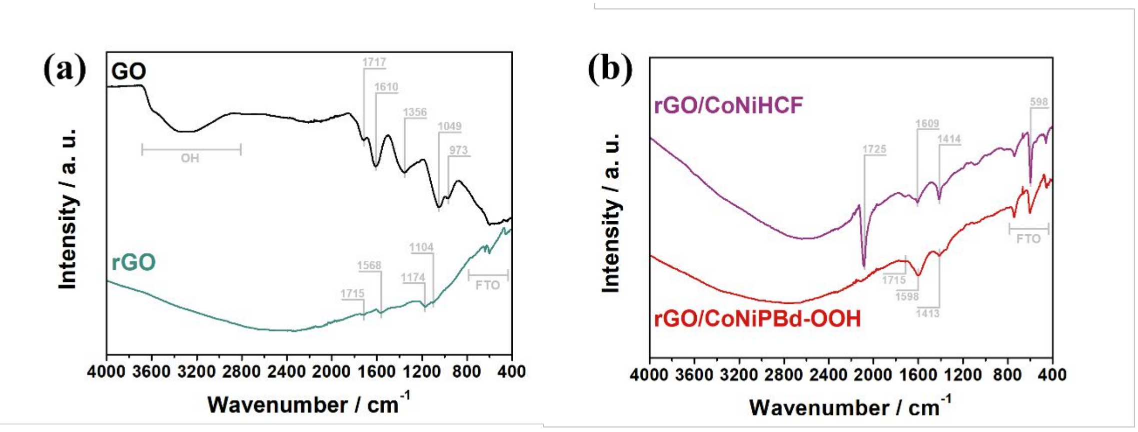

Figure S6. FTIR spectra of the **(a)** GO and rGO, and **(b)** rGOCoNiHCF and rGO/CoNiPBd-OOH-modified FTO electrodes.

Table S1. Parameters obtained by the Raman spectra of the carbon-composed materials.

	Parameter						
Material	A_D/A_G	A_{2D}/A_G	$A_D/A_{D'}$	L_a / nm	η_D / cm^{-2}	L_D / nm	L_{eq} / nm

**GO**	2.78	0.52	6.77	6.92	6.25×1011	7.2	7.16
**rGO**	3.74	0.70	5.62	5.14	8.41×1011	6.21	6.21
**rGOCoNiHCF**	2.81	0.62	5.96	6.85	6.31×1011	7.17	8.45
**rGOCoNiPBd-OOH**	2.78	0.69	5.68	6.92	6.24×1011	7.2	8.45

$$L_a[nm] = (2.4 \times 10^{-10})\ \lambda^4\ (A_D/A_G)^{-1}$$ **Equation S12**

$$\eta_D[cm^{-2}] = \frac{1.8\times10^{22}}{\lambda^4}(A_D/A_G)$$ **Equation S13**

$$L_D[nm] = \sqrt{(1.8 \times 10^{-9})\ \lambda^4\ (A_D/A_G)^{-1}}$$ **Equation S14**

$$L_{eq}[nm] = 38.4492\ (A_{2D}/A_D)$$ **Equation S15**

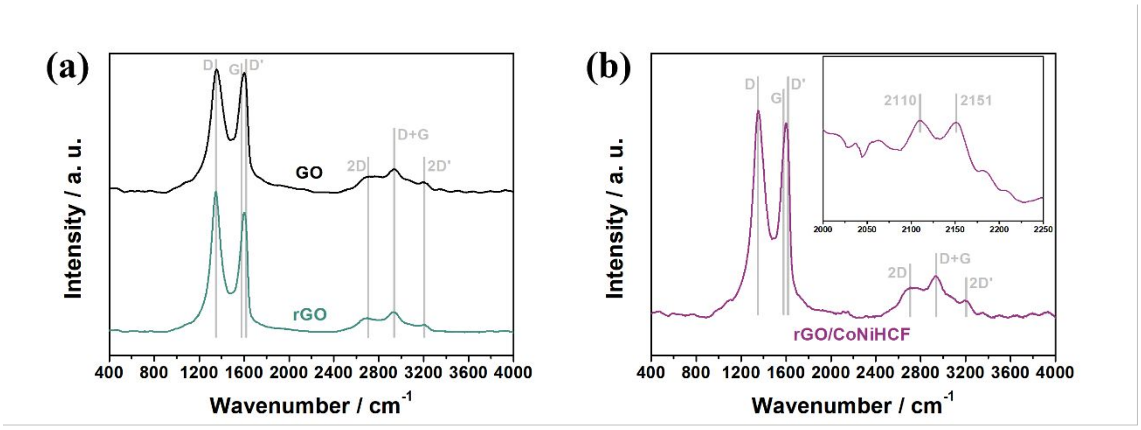

Figure S7. Raman spectra of the **(a)** GO and rGO, and **(b)** rGOCoNiHCF-modified FTO electrodes.

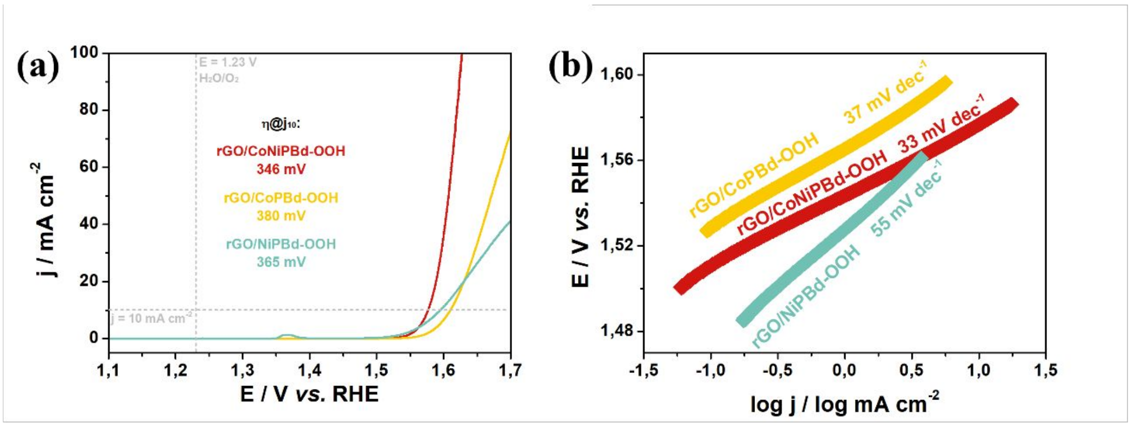

Figure S8. **(a)** LSV in 1.0 mol L^{-1} KOH solution at 5 mV s^{-1} of bimetallic rGO/CoNiPBd-OOH and monometallic rGO/CoPBd-OOH and rGO/NiPBd-OOH. **(b)** Tafel slope of the OER electrocatalysis region promoted by the materials.

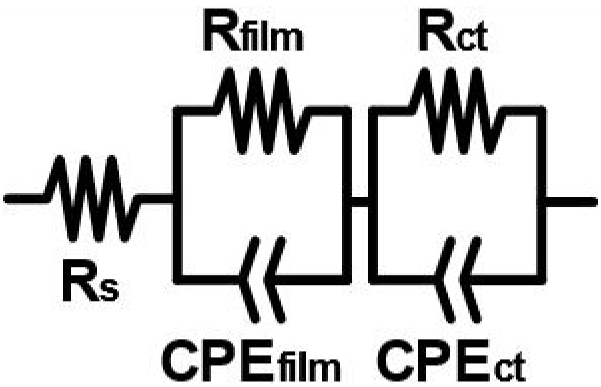

Figure S9. EIS equivalent circuit.

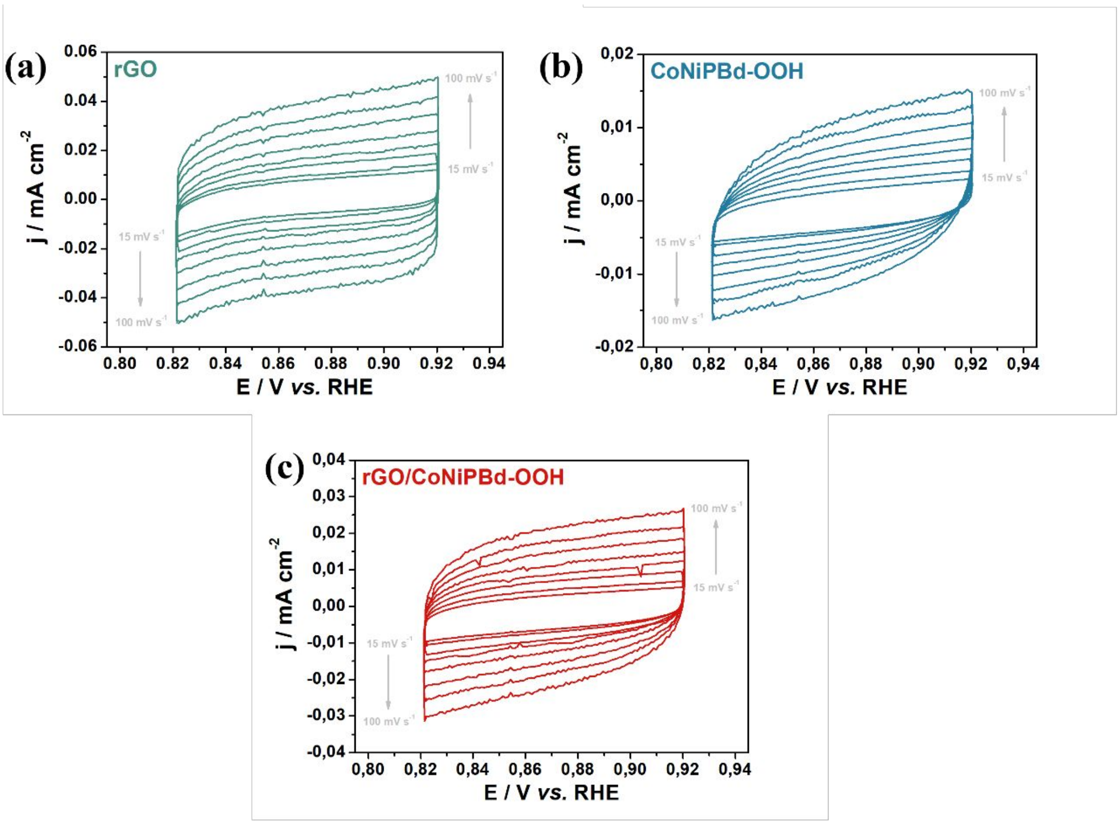

Figure S10. CV of the electrocatalysts in 1.0 mol L^{-1} KOH solution in a non-faradaic region at different scan rates.

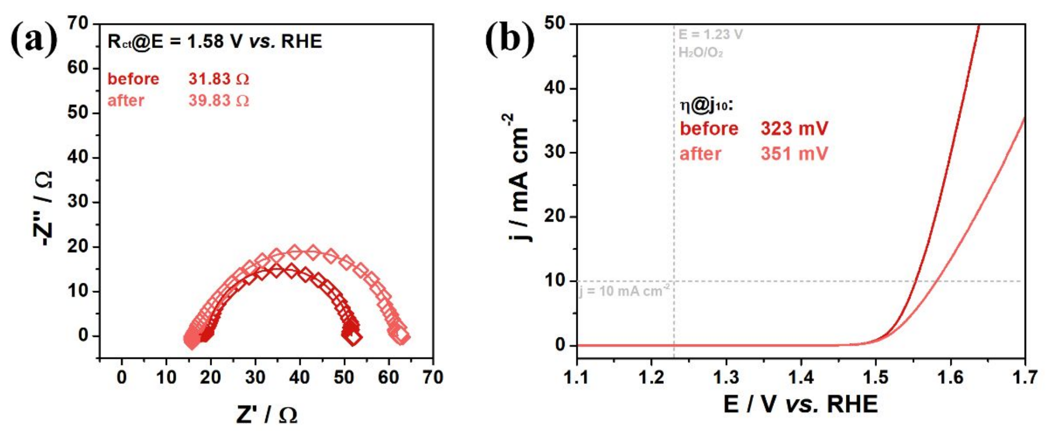

Figure S11. (a) EIS Nyquist diagram and **(b)** LSV at 5 mV s^{-1} in 1.0 mol L^{-1} KOH solution before and after 15 h of stability test.

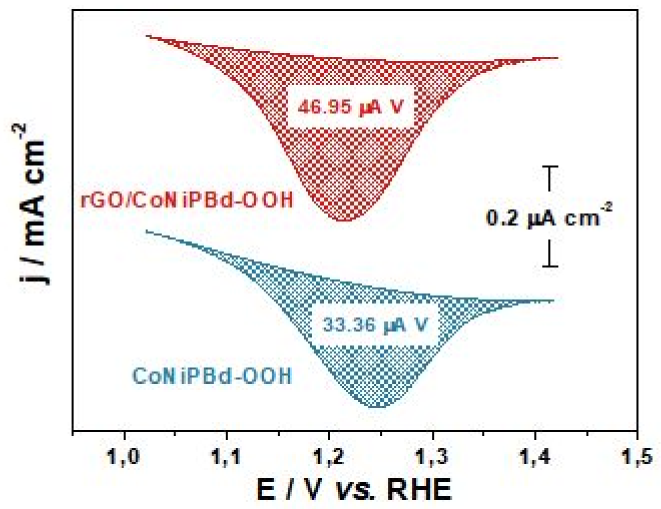

Figure S12. Electrocatalysts CV cathodic peak area integration.
